# Sociodemographic Factors Associated With Using eHealth for Information Seeking in the United States: Cross-Sectional Population-Based Study With 3 Time Points Using Health Information National Trends Survey Data

**DOI:** 10.2196/54745

**Published:** 2024-08-14

**Authors:** Christian Elias Vazquez, Rebecca L Mauldin, Denise N Mitchell, Faheem Ohri

**Affiliations:** 1 School of Social Work The University of Texas at Arlington Arlington, TX United States; 2 Department of Sociology University of North Carolina Chapel Hill, NC United States

**Keywords:** health information seeking, eHealth use, disparities, sex, age, education, mobile phone

## Abstract

**Background:**

Despite the potential benefits of using eHealth, sociodemographic disparities exist in eHealth use, which threatens to further widen health equity gaps. The literature has consistently shown age and education to be associated with eHealth use, while the findings for racial and ethnic disparities are mixed. However, previous disparities may have narrowed as health care interactions shifted to web-based modalities for everyone because of the COVID-19 pandemic.

**Objective:**

This study aims to provide an updated examination of sociodemographic disparities that contribute to the health equity gap related to using eHealth for information seeking using 3 time points.

**Methods:**

Data for this study came from the nationally representative 2018 (n=3504), 2020 (n=3865), and 2022 (n=6252) time points of the Health Information National Trends Survey. Logistic regression was used to regress the use of eHealth for information seeking on race and ethnicity, sex, age, education, income, health status, and year of survey. Given the consistent association of age with the dependent variable, analyses were stratified by age cohort (millennials, Generation X, baby boomers, and silent generation) to compare individuals of similar age.

**Results:**

For millennials, being female, attaining some college or a college degree, and reporting an annual income of US $50,000-$74,999 or >US $75,000 were associated with the use of eHealth for information seeking. For Generation X, being female, having attained some college or a college degree, reporting an annual income of US $50,000-$74,999 or >US $75,000, better self-reported health, and completing the survey in 2022 (vs 2018; odds ratio [OR] 1.80, 95% CI 1.11-2.91) were associated with the use of eHealth for information seeking. For baby boomers, being female, being older, attaining a high school degree, attaining some college or a college degree, reporting an annual income of US $50,000-$74,999 or >US $75,000, and completing the survey in 2020 (OR 1.56, 95% CI 1.15-2.12) and 2022 (OR 4.04, 95% CI 2.77-5.87) were associated with the use of eHealth for information seeking. Among the silent generation, being older, attaining some college or a college degree, reporting an annual income of US $50,000-$74,999 or >US $75,000, and completing the survey in 2022 (OR 5.76, 95% CI 3.05-10.89) were associated with the use of eHealth for information seeking.

**Conclusions:**

Baby boomers may have made the most gains in using eHealth for information seeking over time. The race and ethnicity findings, or lack thereof, may indicate a reduction in racial and ethnic disparities. Disparities based on sex, education, and income remained consistent across all age groups. This aligns with health disparities literature focused on individuals with lower socioeconomic status, and more recently on men who are less likely to seek health care compared to women.

## Introduction

### Background

When paired with appropriate eHealth literacy, using eHealth can help improve individuals’ management of their health [1,2]. eHealth has been defined as the use of information and communication technologies for health and can include the use of email, SMS text messaging, websites, and mobile apps [3]. eHealth use has been associated with better physical and mental health, healthier behaviors, fewer health risk factors, knowledge of medical issues, and more informed health care decision-making [4-7]. Despite its potential benefits, some groups are less likely to adopt eHealth and as a result may experience related health or health care disparities.

It is well documented both within and outside of the United States across time that older adults use eHealth at lower rates than younger adults [8-10]. However, regarding eHealth use among racial and ethnic groups, the literature has produced mixed findings in recent years [9,11,12]. Even in the few studies that use nationally representative data from the United States, the association of race and ethnicity with eHealth use is inconsistent [10,12-15].

A study from 2013 using data from California found that using eHealth for information seeking was significantly lower for Hispanic, Black, and Asian adults than White adults [16]. In a 2014 nationally representative sample of 1336 US residents aged ≥54 years, White respondents were more likely to report using eHealth for information seeking than Black or Hispanic respondents [17]. However, for adults aged ≥75 years, there were no significant racial or ethnic differences in using eHealth for information seeking. For younger adults in this same sample, there were no ethnic or racial differences in using eHealth for information seeking (eg, SMS text messages, mobile apps, and emails), but significant differences emerged beginning at the age of 62 years [17]. These findings indicate that racial and ethnic differences in using eHealth for information seeking may be most appropriately examined within subgroups by age.

The literature also shows significant trends in other key sociodemographic characteristics that are critical to health equity. In addition to age, race, and ethnicity, a recent meta-analysis of studies examining correlates of using eHealth for information seeking identified income, education, sex, and health status as key characteristics to monitor [18]. Income and education were both positively associated with eHealth use, while better self-reported health and being female were also associated with eHealth use, and this is commonly reported both within and outside of the United States [18-23]. A previous study using the same data as this study, Health Information National Trends Survey (HINTS), from 2008 to 2017, found that using eHealth for information seeking was associated with an annual household income >US $20,000 [15]. The health status finding only appears to be significant in nonrepresentative samples, further suggesting that previous findings must be reproduced with representative samples [18]. To support efforts to identify and reduce health disparities across a variety of domains, regularly monitoring these factors in addition to age and race and ethnicity as potential correlates to eHealth use is important.

It is acknowledged that using eHealth for information seeking alone may not necessarily lead to being able to manage or improve health; a common issue is an inability to identify misinformation or understand the information received (ie, having insufficient eHealth literacy) [1,2]. However, systematic reviews of cross-sectional studies and independent intervention studies provide evidence that prior eHealth use is one of the strongest predictors of eHealth literacy mastery [18,24,25]. Using eHealth should precede eHealth literacy training or happen in tandem, which has been effective with a diverse set of participants [24,25]. eHealth literacy is associated with positive outcomes such as the identification of accurate information and better health management (ie, adherence) [26,27]. Thus, identifying those who are less likely to use eHealth—groups that historically may have been excluded from achieving health equity—and targeting them for intervention is critical to address health equity, given the potential benefits that effective eHealth use can have for individuals’ health.

### This Study

As eHealth continues to evolve at a rapid pace, identification of disparities must be kept up to date to every few years, helping interventionists and practitioners understand which groups may need help to maximize the benefits of eHealth. This study will (1) provide updated evidence of sociodemographic disparities across these domains related to the use of eHealth for information seeking in the United States using the most recent publicly available nationally representative relevant data at the time of analysis and (2) use 3 time points to provide evidence of possible growth in eHealth use over time, stratified by adult age cohorts (ie, millennials, Generation X, baby boomers, and silent generation).

In addition to the literature indicating that results may vary by age group, the literature on each generation’s experiences with technology adds to the rationale for stratifying by age cohort. For example, baby boomers and the silent generation grew up without the internet, Generation X is considered the first technologically literate generation, and millennials were teenagers when the internet was launched [28,29]. Thus, these 4 generations had dramatically different experiences with computers, internet, and technology. eHealth interventionists who work with older adults document the need of tailoring interventions based on age, given the relationship between age and prior experience with technology [24,25].

The literature has documented an increase in eHealth use since 2008 [12,15,18]. Though statistically significant changes are not seen yearly or even biennially, they are typically seen over longer periods and have increased only steadily. Given the large shift in how people interacted with health care or accessed health care information due to the COVID-19 pandemic, it is expected that eHealth use has increased in recent years; however, it is essential for public health science to document this with evidence. The last update with multiple time points of nationally representative data used data up to 2017. Thus, this is the rationale for using 2018 as the baseline for this study. The expected evidence showing increases in eHealth use could provide further justification for studies continuing to refine eHealth use engagement and literacy.

This study adds to the literature in several ways. First, it uses the most recent (at the time of analysis) publicly available nationally representative data that asks about eHealth use behaviors. Second, while some studies have used multiple data time points, to our knowledge, no studies to date have examined sociodemographic differences within multiple adult age cohorts with multiple data time points. Third, the pooling of data from 2018, 2020, and 2022 provides evidence across multiple time points to monitor health disparities in using eHealth for information seeking. Collectively, these contributions may help in the identification of groups to target for interventions that assist individuals to maximize the benefits of eHealth.

### Research Questions

This study is driven by two overarching research questions. Research question 1: Aggregating 3 time points, what sociodemographic factors are associated with using eHealth for information seeking in the United States, stratified by age cohort? Research question 2: Do the data indicate that individuals within each age cohort are, on average, more likely to use eHealth for information seeking in more recent time points (eg, 2018 vs 2020 vs 2022), controlling for key sociodemographic factors?

## Methods

### Sample and Data Collection

Data for this study came from 3 time points (2018, 2020, and 2022) of the HINTS. HINTS is an assessment of the American public’s access to and use of information about cancer across the cancer care continuum, spanning from prevention and early detection to diagnosis, treatment, and survivorship. In addition to this standard HINTS content, each cycle also includes questions relating to general eHealth information-seeking behaviors not specific to cancer information. HINTS is a nationally representative survey of the noninstitutionalized adult population (aged >18 years) in the United States, conducted every few years since 2003, and from 2020, it is consistently administered biennially [30-32]. Respondents were sampled using a complex sampling design to achieve a nationally representative sample. Data collection for 2018 started on January 26, 2018, and concluded on May 2, 2018 (n=3504), and had a response rate of 32.9%. Data collection for 2020 started on February 24, 2020, and concluded on June 15, 2020 (n=3865), and had a response rate of 37%. Data collection for 2022 started on March 7, 2022, and concluded on November 8, 2022 (n=6252), and had a response rate of 28.1%. Data were collected using a self-administered survey sent by mail for 2018 and 2020. For 2022, two different data collection modes were available: self-administered web-based surveys and self-administered surveys sent by mail. Surveys were available in English and Spanish. More information about the design, procedures, measures, and weighting of HINTS cycles can be found elsewhere [30-32]. Final analytic samples by time point were n=2612 for 2018, n=2962 for 2020, and n=4971 for 2022.

### Ethical Considerations

Information about informed consent and participant compensation from original HINTS data collection periods are published elsewhere [30-32]. The original data collection was approved by the Westat institutional review board. Ethics review and approval for this analysis were waived due to the study being a secondary analysis of deidentified publicly available data [33]. The authors registered with the HINTS website to use the publicly available data.

### Variables

#### Dependent Variable

The dependent variable conceptually measured whether the respondents used eHealth for information seeking in the past 12 months. This was operationalized by creating a dichotomous variable based on the respondent’s answer to 3 yes or no questions that (1) captured various types of eHealth information seeking and (2) were consistent across the 3 time points. The final variable was coded 1 if they answered yes to any of the 3 questions and 0 if they answered no to all questions. There is a caveat with the abovementioned reason number 2 because the question changed slightly in 2022; however, the researchers of this study suggest the changes capture the same concept, and this is also stated as a limitation. The questions for 2018 and 2020 asked: “In the past 12 months, have you used a computer, smartphone, or other electronic means to do any of the following?” (1) “Looked for health or medical information for yourself?” (2) “Used email or the internet to communicate with a doctor or doctor’s office?” and (3) “Looked up medical test results.” For 2022, the question asked: “In the past 12 months, have you used the internet to take care of any of the following health-related needs?” (1) “Look for health or medical information”; (2) “Send a message to a health care provider or a health care provider’s office”; and (3) “View medical test results.” The response options were no (2) and yes (1).

#### Independent Variables

##### Sex

The sex question asked respondents to report their gender at birth as either male or female. Although the item uses the term “gender,” we believed it aligned more closely with our conceptualization of sex, based on the wording and response options.

##### Age Cohort

Given the consistent and well-documented association between older age and reduced use of eHealth for information seeking, this study stratified analyses by age cohort to assess differences among respondents of similar age. The age cohorts of interest were millennials, Generation X, baby boomers, and silent generation. Respondents were grouped based on cohort ages defined by the Pew Research Center [34]. The age range for millennials in 2018 was 22 to 37 years, in 2020 was 24 to 39 years, and in 2022 was 26 to 41 years. The age range for Generation X in 2018 was 38 to 53 years, in 2020 was 40 to 55 years, and in 2022 was 42 to 57 years. The age range for baby boomers in 2018 was 54 to 72 years, in 2020 was 56 to 74 years, and in 2022 was 58 to 76 years. The age range for the silent generation in 2018 was 73 to 89 years, in 2020 was 75 to 91 years, and in 2022 was 77 to 93 years. Age was also included as a continuous variable within each stratified analysis. Gen Z was also present in the data; however, they were excluded from the sample given that a portion of this group was of college age and may not have had a chance to complete college or enter the workforce full time, conflicting with 2 other key sociodemographic factors (ie, education and income).

##### Race and Ethnicity

The analysis included all racial and ethnic groups in the sample, including those who identified as Asian, Black, Hispanic, other, and White. White was the reference group. The Asian group included those identifying as Asian Indians, Chinese, Filipino, Japanese, Korean, Vietnamese, and other Asian. The Hispanic group included those identifying as Mexican, Puerto Rican, Cuban, other Hispanic, and multiple Hispanic ethnicities. The “other” group included those identifying as Native Hawaiian, Guamanian or Chamorro, Samoan, or other Pacific Islander.

##### Education

Education was originally coded as 4 categories (less than high school, high school graduate, some college, and college graduate). Three dummy coded variables were created with less than high school as the reference.

##### Annual Income

The data included an income variable with 5 categories, including <US $20,000, US $20,000-$34,999, US $35,000-$49,999, US $50,000-$74,999, and ≥US $75,000. Dummy codes were created with <US $20,000 as the reference. This variable was kept in this form because it was used in the same way in a previous study using HINTS data, with eHealth information-seeking behaviors as the outcome [15].

##### Health Status

The general health question asked, “In general, would you say your health is...” and was self-reported as “excellent,” “very good,” “good,” “fair,” and “poor.” This was used as a continuous variable and recoded with poor=1 and excellent=5.

##### Year

The year of survey was included to determine differences between time points. This was coded as 1=2018, 2=2020, and 3=2022.

### Statistical Analyses

The analyses were performed using SAS (version 9.3; SAS Institute) [35]. Complete case analysis was used. Thus, only cases without missing data for all included variables were used. This study used PROC SURVEYLOGISTIC with replicate weights and the jackknife approach as suggested by the HINTS documentation to provide population-level results adjusted for the sampling methods [30-32]. Because we combined 3 time points to test for trends across time points, we created a new set of 150 replicate weights (50 for each time point) using the Rizzo method [36]. Sample descriptive characteristics were examined using frequencies, percentages, means, and SDs. This was done with PROC SURVEYFREQ and SURVEYMEANS with the final sample weight. Chi-square and ANOVA tests were also conducted to assess potential differences of variables across time points.

This study used binary logistic regressions stratified by 4 age cohorts to examine sociodemographic associations with eHealth use for information seeking and to assess differences in use across the years 2018, 2020, and 2022. All variables of interests were added to the model. The reference categories were White race, male sex, less than high school graduate education level, annual income <US $20,000, and the year 2018. The 4-category age cohort variable was included in the DOMAIN option in SAS for stratification. The results for the full sample were also reported for a snapshot of the pooled time points 2018, 2020, and 2022. Odds ratios (ORs) and CIs were used to assess significance.

## Results

### Descriptive Statistics

Table 1 shows the descriptive characteristics of the study sample as well as *P* value for tests of differences across time points. Slightly more female respondents competed the survey in 2022 (2484/4188, 51.45%) compared to 2020 (1651/2927, 50.58%) and 2018 (1478/2576, 49.18%; *P*=.04). The tests of differences resulted in a *P*=.02; however, the order of largest to smallest groups remained the same: White (largest), Hispanic, Black, Asian, and other (smallest). For education, there was a statistically significant difference across time points (*P*<.001), and the order of largest to smallest groups also remained the same: some college, college graduate, high school graduate, and less than high school. The pattern of similar ordering was also evident with the income variable: ≥US $75,000, US $50,000-$74,999, <US $20,000, US $35,000-$49,999, and US $20,000-$34,999, where a significant difference was also observed across time points (*P*<.001). There was not a statistically significant difference in self-reported health status across time points (*P*=.06), with the average rating being “good” health. There was a significant difference in the size of cohort groups across time point (*P*≤.001), but the order of the size of the groups remained the same: Generation X, baby boomers, millennials, and silent generation. There was a significantly (*P*≤.001) smaller proportion of respondents who reported using eHealth in the past 12 months in 2018 and 2020 (1993/2576, 78.89% and 2386/2927, 82.38%) compared to 2022 (3877/4188, 91.31%). When examining differences in eHealth use among age cohorts within each time point, the proportion of respondents who reported using eHealth decreased with increasing age across all time points (*P*≤.001 for all 3 time points, not shown in Table 1).

**Table 1:** Descriptive characteristics of the study sample from the Health Information National Trends Survey for 2018, 2020, and 2022.

Characteristics			Full sample (N=9691)		2018 (n=2576)		2020 (n=2927)		2022 (n=4188)		*P* value (chi-square or ANOVA)	
**Sex, n (%)**												.04
	Male	4078 (49.62)^a^		1098 (50.82)		1276 (49.41)		1704 (48.55)				
	Female	5613 (50.38)		1478 (49.18)		1651 (50.58)		2484 (51.45)				
**Age (years), n (%); mean (SD)**												<.001
	Millennials^b^	2035 (28.36); 32.7 (4.60)		443 (25.88); 30.6 (4.31)		559 (28.16); 32.1 (4.37)		1033 (31.25); 34.9 (4.42)				
	Generation X^c^	2595 (37.08); 48.4 (4.97)		681 (38.64); 46.1 (4.70)		760 (36.11); 48.0 (4.75)		1154 (36.5); 50.0 (4.69)				
	Baby boomers^d^	4079 (27.97); 65.0 (5.27)		1080 (27.52); 63.3 (5.01)		1296 (28.85); 64.8 (5.15)		1703 (27.46); 66.3 (5.19)				
	Silent generation^e^	982 (6.59); 80.0 (4.46)		372 (7.96); 78.8 (4.64)		312 (6.88); 70.1 (4.31)		298 (4.79); 81.4 (3.93)				
**Race and ethnicity, n (%)**												.02
	Asian	474 (4.99)		108 (4.52)		140 (5.44)		226 (4.99)				
	Black	1363 (10.86)		346 (10.68)		389 (11.37)		628 (10.49)				
	Hispanic	1487 (14.82)		365 (15.9)		479 (14.68)		643 (13.82)				
	White	6019 (65.77)		1652 (65.85)		1820 (65.85)		2547 (65.58)				
	Other	348 (3.55)		105 (3.04)		99 (2.65)		144 (5.11)				
**Education, n (%)**												<.001
	Less than high school graduate	499 (6.42)		165 (8.32)		181 (6.50)		153 (4.29)				
	High school graduate	1484 (19.69)		419 (20.1)		486 (20.73)		579 (18.07)				
	Some college	2805 (38.53)		760 (38.26)		850 (38.60)		1195 (38.75)				
	College graduate	4903 (35.36)		1232 (33.32)		1410 (34.17)		2261 (38.89)				
**Income (US $), n (%)**												<.001
	<20,000	1360 (12.54)		426 (14.82)		438 (12.66)		496 (9.95)				
	20,000-34,999	1205 (10.81)		351 (11.76)		366 (10.95)		488 (9.63)				
	35,000-49,999	1278 (12.26)		341 (12.82)		397 (12.78)		540 (11.07)				
	50,000-74,999	1746 (18.28)		486 (18.21)		510 (18.29)		750 (18.34)				
	≥75,000	4102 (46.12)		972 (42.38)		1216 (45.32)		1914 (51.01)				
Health status, mean (SD)			3.42 (0.94)		3.45 (0.95)		3.44 (0.94)		3.40 (0.93)		.06	
**eHealth use in the past year, n (%)^f^**												<.001
	No	1435 (15.99)		583 (21.11)		541 (17.62)		311 (8.69)				
	Yes	8256 (84.01)		1993 (78.89)		2386 (82.38)		3877 (91.31)				

^a^Values were estimated using final sample weights to account for complex sampling.

^b^The age range for millennials in 2018 was 22 to 37 years, in 2020 was 24 to 39 years, and in 2022 was 26 to 41 years.

^c^The age range for Generation X in 2018 was 38 to 53 years, in 2020 was 40 to 55 years, and in 2022 was 42 to 57 years.

^d^The age range for baby boomers in 2018 was 54 to 72 years, in 2020 was 56 to 74 years, and in 2022 was 58 to 76 years.

^e^The age range for the silent generation in 2018 was 73 to 89 years, in 2020 was 75 to 91 years, and in 2022 was 77 to 93 years.

^f^eHealth use in the past year was coded as yes if the respondent answered yes to one of the three questions asking about using internet and devices to (1) look up health information, (2) communicate with health care provider, and (3) look up medical test results.

### Pooled Sample

The pooled sample is intended to provide a snapshot of the average of the 3 time points. Among the pooled sample, women had higher odds of using eHealth for information seeking compared to men (OR 1.70, 95% CI 1.41-2.05). Older age was associated with lower odds of using eHealth for information seeking (OR 0.97, 95% CI 0.97-0.98). Compared to respondents with less than a high school education, respondents with some college (OR 2.64, 95% CI 1.81-3.85) or a college degree (OR 5.26, 95% CI 3.58-7.72) had higher odds of using eHealth for information seeking. Compared to an annual household income of <US $20,000, reporting an annual income of US $35,000-$49,999 (OR 1.55, 95% CI 1.09-2.21), US $50,000-$74,999 (OR 2.62, 95% CI 1.94-3.54), or ≥US $75,000 (OR 3.60, 95% CI 2.49-5.19) was associated with higher odds of using eHealth for information seeking. Better self-reported health status was associated with lower odds of using eHealth for information seeking (OR 0.82, 95% CI 0.74-0.92). Compared to respondents who completed the survey in 2018, respondents who completed the survey in 2020 (OR 1.26, 95% CI 1.03-1.55) or 2022 (OR 2.59, 95% CI 1.99-3.36) had higher odds of using eHealth for information seeking.

### Millennials

Table 2 provides the logistic regression results for using eHealth for information seeking for the pooled sample and by each age cohort. Among millennials, women had higher odds of using eHealth for information seeking compared to men (OR 2.35, 95% CI 1.32-4.16). Compared to respondents with less than a high school education, respondents with some college (OR 4.63, 95% CI 1.67-12.84) or a college degree (OR 5.39, 95% CI 1.73-16.78) had higher odds of using eHealth for information seeking. Compared to an annual household income of <US $20,000, reporting an annual income of US $50,000-$74,999 (OR 3.75, 95% CI 1.47-9.56) or ≥US $75,000 (OR 5.55, 95% CI 1.79-17.22) was associated with higher odds of using eHealth for information seeking.

**Table 2:** Logistic regression for using eHealth for information seeking, stratified by age cohort, Health Information National Trends Survey for 2018 (n=2576), 2020 (n=2927), and 2022 (n=4188).

	Pooled sample, OR^a^ (95% CI)	Millennials^b^, OR (95% CI)	Generation X^c^, OR (95% CI)	Baby boomers^d^, OR (95% CI)	Silent generation^e^, OR (95% CI)
Female^f^	*1.70 (1.41-2.05)^g^*	*2.35 (1.32-4.16)*	*1.79 (1.17-2.73)*	*1.68 (1.32-2.13)*	1.38 (0.81-2.33)
Age	*0.97 (0.97-0.98)*	1.03 (0.94-1.12)	0.97 (0.93-1.01)	*0.96 (0.93-0.99)*	*0.93 (0.89-0.99)*
Asian^h^	0.85 (0.51-1.40)	0.90 (0.26-3.06)	0.87 (0.39-1.93)	0.80 (0.37-1.74)	1.02 (0.23-4.53)
Black^h^	0.97 (0.69-1.38)	1.00 (0.31-3.28)	1.01 (0.58-1.77)	0.83 (0.57-1.20)	1.17 (0.61-2.23)
Hispanic^h^	0.95 (0.70-1.30)	0.98 (0.45-2.14)	0.89 (0.52-1.53)	0.80 (0.54-1.17)	1.69 (0.76-3.78)
Other^h^	1.37 (0.78-2.39)	0.98 (0.25-3.89)	1.68 (0.54-5.18)	1.22 (0.56-2.65)	2.21 (0.35-14.11)
High school graduate^i^	1.29 (0.86-1.92)	1.14 (0.39-3.34)	1.11 (0.58-2.12)	*2.23 (1.25-3.99)*	1.12 (0.42-2.99)
Some college^i^	*2.64 (1.81-3.85)*	*4.63 (1.67-12.84)*	*2.00 (1.07-3.72)*	*3.23 (1.79-5.84)*	*2.77 (1.11-6.88)*
College graduate^i^	*5.26 (3.58-7.72)*	*5.39 (1.73-16.78)*	*5.32 (2.60-10.89)*	*7.70 (3.96-14.98)*	*3.76 (1.28-11.03)*
US $20,000-$34,999^j^	1.38 (1.00-1.90)	2.49 (0.98-6.31)	1.24 (0.64-2.40)	1.26 (0.81-1.96)	1.35 (0.68-2.70)
US $35,000-$49,999^j^	*1.55 (1.09-2.21)*	2.19 (0.69-7.00)	1.44 (0.79-2.64)	1.35 (0.83-2.21)	1.62 (0.76-3.47)
US $50,000-$74,999^j^	*2.62 (1.94-3.54)*	*3.75 (1.47-9.56)*	*4.63 (2.45-8.76)*	*1.54 (1.01-2.34)*	*2.63 (1.25-5.51)*
≥US $75,000^j^	*3.60 (2.49-5.19)*	*5.55 (1.79-17.22)*	*4.09 (2.24-7.46)*	*2.71 (1.69-4.34)*	*2.92 (1.30-6.58)*
Health status	*0.82 (0.74-0.92)*	0.74 (0.53-1.02)	*0.67 (0.54-0.84)*	0.94 (0.81-1.09)	1.08 (0.83-1.40)
2020^k^	*1.26 (1.03-1.55)*	0.65 (0.32-1.31)	1.45 (0.93-2.27)	*1.56 (1.15-2.12)*	1.37 (0.87-2.14)
2022^k^	*2.59 (1.99-3.36)*	1.42 (0.68-2.94)	*1.80 (1.11-2.91)*	*4.04 (2.77-5.87)*	*5.76 (3.05-10.89)*

^a^OR: odds ratio.

^b^The age range for millennials in 2018 was 22 to 37 years, in 2020 was 24 to 39 years, and in 2022 was 26 to 41 years.

^c^The age range for Generation X in 2018 was 38 to 53 years, in 2020 was 40 to 55 years, and in 2022 was 42 to 57 years.

^d^The age range for baby boomers in 2018 was 54 to 72 years, in 2020 was 56 to 74 years, and in 2022 was 58 to 76 years.

^e^The age range for the silent generation in 2018 was 73 to 89 years, in 2020 was 75 to 91 years, and in 2022 was 77 to 93 years.

^f^Reference category: male (sex).

^g^Statistically significant values are italicized.

^h^Reference category: White (race and ethnicity).

^i^Reference category: less than high school graduate (education).

^j^Reference category: <US $20,000 (income).

^k^Reference category: 2018 (year).

### Generation X

Among Generation X, women had higher odds of using eHealth for information seeking compared to men (OR 1.79, 95% CI 1.17-2.73). Compared to respondents with less than a high school education, respondents with some college (OR 2.00, 95% CI 1.07-3.72) or a college degree (OR 5.32, 95% CI 2.60-10.89) had higher odds of using eHealth for information seeking. Compared to an annual household income of <US $20,000, reporting an annual income of US $50,000-$74,999 (OR 4.63, 95% CI 2.45-8.76) or ≥US $75,000 (OR 4.09, 95% CI 2.24-7.46) was associated with higher odds of using eHealth for information seeking. Better self-reported health status was associated with lower odds of using eHealth for information seeking (OR 0.67, 95% CI 0.54-0.84). Compared to respondents who completed the survey in 2018, respondents who completed the survey in 2022 (OR 1.80, 95% CI 1.11-2.91) had higher odds of using eHealth for information seeking.

### Baby Boomers

Among baby boomers, women had higher odds of using eHealth for information seeking compared to men (OR 1.68, 95% CI 1.32-2.13). Older age was associated with lower odds of using eHealth for information seeking (OR 0.96, 95% CI 0.93-0.99). Compared to respondents with less than a high school education, respondents with a high school education (OR 2.23, 95% CI 1.25-3.99), some college (OR 3.23, 95% CI 1.79-5.84), or a college degree (OR 7.70, 95% CI 3.96-14.98) had higher odds of using eHealth for information seeking. Compared to an annual household income of <US $20,000, reporting an annual income of US $50,000-$74,999 (OR 1.54, 95% CI 1.01-2.34) or ≥US $75,000 (OR 2.71, 95% CI 1.69-4.34) was associated with higher odds of using eHealth for information seeking. Compared to respondents who completed the survey in 2018, respondents who completed the survey in 2020 (OR 1.56, 95% CI 1.15-2.12) or 2022 (OR 4.04, 95% CI 2.77-5.87) had higher odds of using eHealth for information seeking.

### Silent Generation

Among the silent generation, older age was associated with lower odds of using eHealth for information seeking (OR 0.93, 95% CI 0.89-0.99). Compared to respondents with less than a high school education, respondents with some college (OR 2.77, 95% CI 1.11-6.88) or a college degree (OR 3.76, 95% CI 1.28-11.03) had higher odds of using eHealth for information seeking. Compared to an annual household income of <US $20,000, reporting an annual income of US $50,000-$74,999 (OR 2.63, 95% CI 1.25-5.51) or ≥US $75,000 (OR 2.92, 95% CI 1.30-6.58) was associated with higher odds of using eHealth for information seeking. Compared to respondents who completed the survey in 2018, respondents who completed the survey in 2022 (OR 5.76, 95% CI 3.05-10.89) had higher odds of using eHealth for information seeking.

## Discussion

### Principal Findings

This study set out to (1) provide updated evidence on sociodemographic disparities across these domains related to the use of eHealth for information seeking in the United States using the most recent publicly available nationally representative relevant data at the time of analysis and (2) use 3 time points to provide evidence of possible growth in eHealth use over time, stratified by adult age cohorts, at the population level. eHealth and its use are continuously evolving given the growing ubiquity of technology in health care and should be monitored every few years to keep researchers, practitioners, and policy makers up to date. At the time of analysis, the HINTS 2022 was the most up-to-date publicly available nationally representative data set available for reporting eHealth information-seeking behaviors in the United States. These findings are important to help interventionists understand what populations to target and how this may have changed over time. These findings can be used as a reference for future updates.

Given the consistent association between age and eHealth use and each generation’s historical experience with technology, analyses were stratified by age cohort to assess differences among individuals from the same age group. Though being younger has historically been associated with higher eHealth use, the findings of this study highlight sex, education, and income disparities even within the youngest age groups. For Generation X, in addition to the disparities seen in the millennial cohort, those with better self-reported health were also less likely to use eHealth for information seeking. For baby boomers, those who were male, older, had less than a high school education, and reported income <US $50,000 were less likely to use eHealth for information seeking. For the silent generation, those who were older, had less than some college education, and reported income <US $50,000 were less likely to use eHealth for information seeking. The findings from the pooled analysis align with the findings from previous studies with pooled adult population analysis; however, the stratified findings from this study highlight the need to examine the findings by age group [9,11,12,17,18]. For example, no significant differences appear to exist by race and ethnicity for any group, which have been seen in previous work. This study also shows how the educational disparities related to eHealth use are mostly consistent with a slight variation for baby boomers. In addition, the results indicate that baby boomers and silent generation may have made the largest gains in using eHealth for information seeking over time, with Generation X also making gains, which is something that was not observed within the millennial cohort. This is a promising finding for older adults in the United States because it aligns with the literature from other countries, which suggests that older adults are ready to engage with eHealth and eHealth interventions [37].

The education and income findings show strong consistency. Similarly, the sex finding is consistent, with the exception of the silent generation. The lack of statistically significant racial and ethnic differences across all cohorts and time points is consistent in this study that uses nationally representative data; however, there continues to be mixed findings in this area of the literature overall, with some previous studies suggesting that this only exists with older adults and others reporting that significant findings for racial and ethnic differences tend to appear in analyses using nonrepresentative samples [17,18]. Other pooled studies using older HINTS data mostly found Black adults to use eHealth less than White adults [12,15] or that health information seeking was higher for White adults than Hispanic adults [38]. Those studies did not stratify by age and use data before 2018; however, they may help put the findings of this study in the context of time. Thus, the findings of this study indicate that Hispanic and Black adults may have made gains in using eHealth for information seeking compared to White adults. It will be interesting to follow this divide as well as the age disparity that exists in the oldest age cohorts, given that millennials and Generation X both currently use eHealth at higher rates and will have had more time to adjust to eHealth use compared to baby boomers and the silent generation. The health status finding is also interesting because eHealth use has been associated with better health, but as the findings of this study suggest, those with better self-reported health also tend to be less likely to use eHealth for information seeking; though this only appears for the pooled and Generation X samples.

Overall, the millennial cohort had the least number of disparities with the silent generation following them, though these 2 groups were on opposite ends in regard to use across all time points. The largest number of disparities were observed in the baby boomer cohort. The education finding has been one of the most consistent correlates across the literature [9,11,12,17,18]. The result for less than high school education compared to high school graduate for baby boomers was a result that was unique to this group, even when looking at the pooled analysis. Addressing educational disparities is particularly important as eHealth may be most useful to those groups considered potentially vulnerable such as older adults, with respect to technology. The sex findings are concerning as men continue to have lower life expectancy than women, and the literature suggests that men are less likely to seek out health care in general compared to women [39,40]. In the younger age groups, it may also be the case that men have fewer reproductive health information needs than women.

### Implications

We acknowledge earlier literature suggesting that, similar to general internet use, using eHealth for information seeking can have both positives and negatives, with misinformation and scams or fraud being some of the main negatives [1,2]. While this study did not assess misinformation or being scammed or defrauded or eHealth literacy, it is important to note that using eHealth with a sufficient level of eHealth literacy can help address some of these issues. Furthermore, the groups considered most vulnerable may be the most susceptible to misinformation and scams or fraud. This is relevant to this study because researchers who may want to focus on increasing eHealth use among those who are less likely to use it, based on the findings of this study (eg, those with lower education and low income), should take caution to pair any intervention that promotes uptake with appropriate eHealth literacy to help prevent negative outcomes of using eHealth. eHealth literacy interventions have proven to be effective at increasing eHealth literacy up to 61% for a diverse range of individuals [24]. In addition, relevant to this study, interventionists have seen differential dropout rates based on race, ethnicity, sex, and prior computer experience, which led other researchers to develop machine learning–based approaches that can provide useful information for predicting retention [24,41]. Studies with up-to-date population-based data can contribute to this and other types of artificial intelligence approaches to provide initial clues as to whom and how to target with appropriate strategies.

On the positive end, once individuals know how to use eHealth effectively, they can take advantage of technologies that allow health care providers to assist in health management. Adults may upload food logs, blood sugar levels, and drugs taken, which the health care providers can check in real time [41]. Other tools for older adults include ones that can detect changes in daily activities, such as falls, and devices that send notifications for one to exercise or take prescriptions [42]. However, these eHealth tools are only useful if one is using eHealth and is eHealth literate. Thus, using eHealth for information seeking is an ideal place to start this journey. Using eHealth effectively can also help reduce stress, such as navigating a web-based health portal to save time or being able to check a physician’s message on their own time instead of waiting for a phone call. As telehealth becomes more common, when appropriate, individuals can take appointments at home, avoiding travel and schedule conflicts.

Our results may inform health educators when selecting intervention target populations and sites. For example, the consistent finding that those with a high school education or less are less likely to use eHealth for information seeking suggests that intervention at the middle school or high school level could be beneficial. Health education is common in school systems, and adding eHealth information seeking and literacy to the curriculum is relevant and could help reduce the educational disparities in eHealth information seeking. Similarly, sites with older adult classes, groups, and activities such as older adult centers, assisted living communities, and retirement communities could also be valuable locations for eHealth and literacy education.

However, it is important to note that access to internet services and devices is also imperative for eHealth use. Public policies that increase access to broadband or freely available Wi-Fi hot spots could be seen as having public health implications by reducing barriers to eHealth information seeking. Indeed, as a public health intervention, landing pages for free public internet services could be set to default to reputable health information websites as a way to promote eHealth information-seeking behaviors. Similarly, as part of their business practices, technology companies in the private sector could engage in social well-being efforts by expanding access to low-cost internet services and technological devices and pairing these efforts with campaigns that promote eHealth information seeking and literacy. The importance of these efforts was highlighted during the COVID-19 pandemic, as in-person information channels were restricted and those most underserved and underresourced, such as those with characteristics identified in this study, may have benefitted greatly.

### Limitations

A strength of this study is that it uses a nationally representative data set. However, a limitation to using this secondary data was that the measure for eHealth information seeking was a dichotomous composite of 3 items that varied slightly across time points and could have been interpreted differently, particularly in 2022 compared to 2018 and 2020. Furthermore, this variable only partially captures the complex phenomenon of easily accessing health information on the internet. Measures of general literacy, health care literacy, and English proficiency, which may be related to the information seeking experience, were not available in the HINTS data sets that were analyzed. Another potential limitation is bias in survey completion, as those who are most underrepresented and underserved may face barriers that prevent their participation in this type of survey. For example, those who do not speak English or Spanish, those who may be displaced (ie, no physical address) and do not have internet or phone access, and those who may have literacy issues not related to speaking a different language are people who represent some of the most underserved and underrepresented populations in health research. An inevitable limitation of any technology study is that technology evolves rapidly, rendering some components outdated. Continuous updating of intervention components and outcome measures will be necessary for future studies. The HINTS longitudinal data set will allow for future comparisons. It is important to acknowledge that the data collection period for the 2020 data overlapped with the COVID-19 social distancing measures when people were at home and potentially more likely to be using eHealth; however, the proportions of respondents who reported using eHealth in the past 12 months in 2018 and 2020 (1993/2576, 78.89% and 2386/2927, 82.38%) were closer in size compared to 2022 (3877/4188, 91.31%). It is also possible that respondents could have still been practicing certain social distancing measures during part of the 2022 reporting period (ie, in the past 12 months). The findings from this study align with findings from samples in other countries during this period [26,43,44]. It is also important to note that HINTS is a trend study and not a panel study. As such, the observed trends over time do not represent within-respondent changes but rather larger trends in the population as a whole.

### Conclusions

eHealth information seeking is an important step in maximizing eHealth use in general, which promotes individual health and well-being. As technology rapidly evolves, disparities in eHealth information behaviors also shift. Recognizing changing disparities over time is a necessary step for closing gaps in eHealth information seeking for members of groups considered vulnerable or underserved groups and may be an important component in public health efforts to reduce overall health disparities. Results from this study support a growing body of literature that racial and ethnic gaps in eHealth information seeking may be closing. This promising trend provides hope for future efforts to reduce other types of disparities in eHealth information seeking. With regular monitoring and interventions tailored to populations in need, the public health community may also realize success in reducing disparities for adults with lower education levels.

## Abbreviations

HINTS: Health Information National Trends Survey
OR: odds ratio

## Data Availability

The data sets generated during and analyzed during this study are available in the Health Information National Trends Survey repository [45].
